# Raf-like kinases and receptor-like (pseudo)kinase GHR1 are required for stomatal vapor pressure difference response

**DOI:** 10.1073/pnas.2107280118

**Published:** 2021-11-19

**Authors:** Po-Kai Hsu, Yohei Takahashi, Ebe Merilo, Alex Costa, Li Zhang, Klara Kernig, Katie H. Lee, Julian I. Schroeder

**Affiliations:** ^a^Cell and Developmental Biology Section, Division of Biological Sciences, University of California San Diego, La Jolla, CA 92093;; ^b^Institute of Technology, University of Tartu, Tartu 50411, Estonia;; ^c^Department of Biosciences, University of Milan, Milan 20133, Italy;; ^d^Institute of Biophysics, Consiglio Nazionale delle Ricerche, 20133 Milan, Italy

**Keywords:** relative air humidity, vapor pressure deficit, abscisic acid, Raf-like MAP kinase kinase kinases, GUARD CELL HYDROGEN PEROXIDE-RESISTANT1

## Abstract

Stomatal pores close rapidly in response to low-air-humidity-induced leaf-to-air vapor pressure difference (VPD) increases, thereby reducing excessive water loss. The hydroactive signal-transduction mechanisms mediating high VPD–induced stomatal closure remain largely unknown. The kinetics of stomatal high-VPD responses were investigated by using time-resolved gas-exchange analyses of higher-order mutants in guard-cell signal-transduction branches. We show that the slow-type anion channel SLAC1 plays a relatively more substantial role than the rapid-type anion channel ALMT12/QUAC1 in stomatal VPD signaling. VPD-induced stomatal closure is not affected in *mpk12*/*mpk4GC* double mutants that completely disrupt stomatal CO_2_ signaling, indicating that VPD signaling is independent of the early CO_2_ signal-transduction pathway. Calcium imaging shows that osmotic stress causes cytoplasmic Ca^2+^ transients in guard cells. Nevertheless, *osca1-2*/*1.3*/*2.2*/*2.3*/*3.1* Ca^2+^-permeable channel quintuple, *osca1.3*/*1.7*-channel double, *cngc5*/*6*-channel double, *cngc20*-channel single, *cngc19*/*20crispr*-channel double, *glr3.2*/*3.3*-channel double, *cpk-*kinase quintuple, *cbl1*/*4*/*5*/*8*/*9* quintuple, and *cbl2*/*3rf* double mutants showed wild-type-like stomatal VPD responses. A B3-family Raf-like mitogen-activated protein (MAP)-kinase kinase kinase, M3Kδ5/RAF6, activates the OST1/SnRK2.6 kinase in plant cells. Interestingly, B3 Raf-kinase *m3kδ5* and *m3kδ1*/*δ5*/*δ6*/*δ7* (*raf3*/*6*/*5*/*4*) quadruple mutants, but not a 14-gene *raf-kinase* mutant including osmotic stress-linked B4-family Raf-kinases, exhibited slowed high-VPD responses, suggesting that B3-family Raf-kinases play an important role in stomatal VPD signaling. Moreover, high VPD–induced stomatal closure was impaired in receptor-like pseudokinase GUARD CELL HYDROGEN PEROXIDE-RESISTANT1 (GHR1) mutant alleles. Notably, the classical transient “wrong-way” VPD response was absent in *ghr1* mutant alleles. These findings reveal genes and signaling mechanisms in the elusive high VPD–induced stomatal closing response pathway.

## Significance

With the continuing increase in global temperatures, plants transpire more water due to the increasing vapor pressure deficit. Stomatal pores in plants close rapidly in response to the rising vapor pressure deficit to counteract water loss. We demonstrate that mutations in the stomatal CO_2_ signaling pathway do not impair the response to an increase in vapor pressure difference (VPD). Osmotic stress causes cytoplasmic Ca^2+^ transients in guard cells. Nevertheless, we show that diverse investigated higher-order calcium-signaling mutants do not affect the VPD response. We reveal that B3 family Raf-like protein kinases and a plasma membrane receptor-like protein GHR1 function in the elusive leaf-to-air VPD-mediated stomatal closure pathway. Notably, *ghr1* mutant alleles disrupt the classical “wrong-way” stomatal VPD response.

Stomata are located in the epidermis of aerial tissues of plants and consist of a pair of guard cells that bend apart under increased turgor to open a gas-exchange pore. By adjusting their pore apertures in response to abiotic stresses and stimuli, including water status, temperature, and carbon dioxide (CO_2_) concentrations, stomata regulate transpiration and CO_2_ influx into leaves. By influencing the rate of transpiration and hence leaf water status, changes in relative air humidity trigger the regulation of stomatal apertures (1, 2). Lowering the relative humidity in the air surrounding leaves increases the leaf-to-air vapor pressure difference (VPD). This increase in VPD, in turn, results in an initially enhanced transpiration rate, but stomata then respond by partially closing, thereby reducing water loss of plants. Two mechanisms can contribute to high-VPD-triggered stomatal closure:

1. A hydropassive mechanism suggests that the passive equilibration of guard-cell water potential with VPD-induced reduction in external water potential reduces guard-cell turgor and thereby the stomatal pore aperture (3–8).
2. A hydroactive mechanism suggests that the stomatal closing response to increased VPD requires a metabolically and ion-transport-driven/guard-cell signal-transduction-driven reduction in guard-cell solute concentration (2, 5, 7). This involves signal transduction in guard cells, leading to guard-cell ion-channel and transporter regulation, guard-cell ion efflux, and a reduction in guard-cell solutes, resulting in turgor reduction and stomatal closing (2, 5).

These two mechanisms do not exclude one another, and both likely contribute to stomatal closing, depending on the species and conditions (2, 5), with the hydroactive mechanism considered to be of greater importance in angiosperm species with an epidermal mechanical advantage over guard cells, notably in grasses (9–12). However, the molecular mechanisms of VPD sensing and VPD-induced signal transduction during the hydroactive response remain to a large degree unknown.

Slow (S-type) anion channels (13) and rapid (R-type) anion channels (14) play important roles in mediating and regulating guard-cell ion efflux and stomatal closure in response to upstream signaling events. These anion channels mediate anion efflux and cause plasma-membrane depolarization in guard cells (13, 14). The S-type anion channel SLAC1 and the R-type anion channel ALMT12/QUAC1 function in stomatal closure (15–18). Mutants in the *SLAC1* gene show impairment in stomatal closure in response to several environmental stimuli, including abscisic acid (ABA), CO_2_, ozone, and low relative humidity (17, 18).

The plant hormone ABA plays a central role in regulating stomatal closure (19–21). ABA-mediated stomatal regulation evolved 400 million years ago in moss species (22). ABA triggers stomatal closure through direct binding to PYR/PYL/RCAR receptors, which, in turn, leads to inhibition of clade A type 2C protein phosphatases (PP2Cs) (23–25). Inhibition of PP2Cs contributes to the activation of OST1/SnRK2.6 protein kinase (26, 27), and then activated OST1/SnRK2.6 in guard cells stimulates S-type anion channels encoded by *SLAC1* (28–32). The role of ABA in high-VPD-mediated hydroactive stomatal closure has been investigated in several ABA biosynthesis mutants and ABA signaling mutants in *Arabidopsis*. The resulting models have been, to a degree, debated. Early studies on ABA-deficient (*aba1-1*) and ABA-insensitive (*abi1-1* and *abi2-1*) mutants suggested that ABA and ABA signaling are not necessary for high VPD–induced stomatal closure (33). This is supported by the investigation of transgenic lines expressing a guard-cell-targeted dominant *abi1-1* protein phosphatase, which showed a wild-type (WT)-like stomatal response to a rapid increase in VPD, including in time-resolved gas-exchange analyses (34). Overexpression of the *Arabidopsis abi1-1* mutant allele in gray poplar, *Populus* x *canescens* (Aiton) Sm., exhibited a defect in steady-state stomatal conductance in response to a VPD increase (35). In another study, an ABA biosynthesis mutant (*aba2-13*) and an *OST1* protein kinase mutant (*ost1-4*) were identified by genetic screening for mutants defective in low-humidity-mediated steady-state leaf temperature regulation and thus were impaired in low-humidity-mediated stomatal closure (36). It has been proposed that high VPD/low humidity rapidly triggers ABA biosynthesis to induce stomatal closure (37, 38). However, whole-plant gas-exchange analyses showed that stomata close in response to high-VPD stimulation in several ABA-deficient mutants defective in key steps of ABA biosynthesis, including the most severe *nced3*/*nced5* double mutant (5, 39, 40), indicating that a direct increase in the ABA concentration of guard cells may not be essential for initiating high VPD–induced stomatal closure. Furthermore, a step change to low humidity (high VPD) caused a similar percentage degree of reduction in the guard-cell K^+^ content in WT and the ABA biosynthesis *aba3-1* mutant, indicating a functional high-VPD response in the ABA-deficient mutant (37). Moreover, an ABA receptor, *pyr1*/*pyl1*/*pyl2*/*pyl4*/*pyl5*/*pyl8* hextuple mutant, which disrupts ABA-induced stomatal closure (5, 41), exhibited a significantly reduced initial response rate and increased half-response time of stomatal closure during high-VPD stimulation (5). These results indicate that ABA signaling may contribute to the stomatal closing rate of the high-VPD response and steady-state stomatal apertures, but rapid enhancement of ABA signaling is not essential for high VPD–induced stomatal closing to be initiated and can proceed, albeit at a slower rate, in the above mutants (5).

It has been suggested that young leaves acquire stomatal ABA sensitivity by pre-exposure to ABA or low relative humidity for 1 d (42). Analyses of ABA concentration increases in response to drought or low humidity have shown that substantial rises in the ABA concentration appear to occur after 3 or more hours of stress treatment (43), whereas high VPD–induced stomatal closing typically occurs within 15 min. Studies have shown that guard cells in nonstressed leaves have measurable and higher ABA concentrations than mesophyll cells (44–47). Recent studies suggest that steady-state basal ABA signaling occurs in guard cells (46–48), and a model has been proposed that may explain many of the above findings, in which basal ABA signaling may contribute to the stomatal VPD response by amplifying the hydroactive stomatal closure pathway (20), similar to CO_2_-triggered stomatal closure (46, 47). Furthermore, several studies suggest a key role for ABA in adjusting the steady-state stomatal conductance (34, 35). In the present study, we pursued analyses of other guard-cell signal-transduction pathways and higher-order mutants given the limited knowledge of early VPD sensing and signaling mechanisms.

In contrast to the findings of a functional high-VPD response in ABA receptor hextuple mutants, the high-VPD response was strongly impaired in the OST1 protein kinase mutant, *ost1-3* (5), consistent with earlier research (36). It has been suggested that an ABA-independent pathway is involved in OST1/SnRK2 kinase activation during water deficiency (49, 50). However, apart from the requirement of the OST1/SnRK2.6 protein kinase, the molecular and genetic mechanisms that mediate VPD sensing and signal transduction in the rapid hydroactive response remain elusive.

Given the dearth of mutants and mechanisms known to affect the rapid hydroactive VPD-induced stomatal closing response, in the present study, we screened and investigated potential signaling mechanisms to determine mechanisms that mediate the hydroactive stomatal VPD response. We analyzed mutants in several guard-cell signal-transduction pathways to determine which genes, mechanisms, and pathways contribute to the VPD response and which gene combinations alone are not required for the VPD response. The time-dependent kinetics of stomatal VPD responses were investigated by intact leaf gas-exchange analyses in mutants in guard-cell signaling pathways. Differential contributions of the S-type anion channel SLAC1 and the R-type anion channel ALMT12 were found by analyzing the high-VPD response in *slac1-3*, *almt12-1*, and *slac1-3*/*almt12-1* mutants. The involvement of osmotic/mechanical sensing and calcium signaling in the stomatal high-VPD response was investigated in the putative osmotic/mechanical senor *osca1-2*/*1.3*/*2.2*/*2.3*/*3.1* quintuple and pathogen-associated molecular patterns-activated calcium channels *osca1.3*/*1.7* double mutants, glutamate receptor-like channels *glr3.2*/*3.3* double mutant, Ca^2+^-permeable cyclic nucleotide-gated channels *cngc5*/*6* double mutant, *cngc20* and *cngc19*/*20crispr* double mutants, calcium-dependent protein kinase *cpk3*/*5*/*6*/*11*/*23* and *cpk3*/*4*/*5*/*6*/*11* quintuple mutants, and calcineurin-B like proteins *cbl1*/*4*/*5*/*8*/*9* quintuple and *cbl2*/*3rf* double mutants. The stomatal high-VPD responses were also analyzed in strongly CO_2_-insensitive mitogen-activated protein (MAP) kinase *mpk12*/*mpk4GC* double-mutant alleles showing that early CO_2_ signaling does not participate in stomatal VPD signal transduction. Interestingly, mutant alleles in the receptor-like (pseudo)kinase GHR1 greatly impaired the stomatal VPD response and impaired the classical “wrong-way” response to stomata to VPD increases. We further investigated whether the recently identified B3-family and B4-family Raf-like MAP Kinase Kinase Kinases (M3Ks) (51), which function in OST1 activation (52–54), are required for stomatal closure in response to VPD elevation and show that B3-family, but not B4-family, Raf-like M3Ks function in the stomatal VPD response, with a central role for M3Kδ5/RAF6.

## Results

### High VPD–Induced Stomatal Closure Is Slowed in *ost1* Kinase Mutant and Guard-Cell Anion-Channel *slac1* Single and *slac1*/*almt12* Double Mutants.

The OST1/SnRK2.6 protein kinase is an important positive transducer of stomatal closing and an activator of anion channels during stomatal closure. It has been shown that *ost1*/*snrk2.6* mutants are impaired in high VPD–induced stomatal closure (5, 36, 40). In controls, under the imposed experimental conditions, *ost1-3* mutant leaves showed a much higher basal stomatal conductance at low VPD (∼0.8 kPa) compared to WT plants (*SI Appendix*, Fig. S1*A*). Although a transition to elevated VPD (∼2.0 kPa), caused a time-dependent reduction in stomatal conductance in both *ost1-3* mutant and WT leaves, the final stomatal conductances were larger in *ost1-3* mutant leaves, and the time course of the response was consistently slower in independent datasets (*SI Appendix*, Fig. S1*A*). The initial high-VPD stomatal closing responses were fitted with exponential one-phase decay functions. The average rate constant in *ost1-3* mutant leaves was significantly smaller than in WT controls (*SI Appendix*, Fig. S1*B*; *ost1-3*: 0.10 ± 0.01 min^−1^ vs. WT: 0.28 ± 0.06 min^−1^, *P* < 0.03), and the average half-response times were significantly larger in *ost1-3* mutant leaves than WT (*SI Appendix*, Fig. S1*C*; *ost1-3*: 8.9 ± 0.6 min vs. WT: 5.1 ± 0.4 min, *P* < 0.002). These results suggest that high VPD–induced stomatal closure in *ost1-3* was slower than in WT, consistent with previous research (5, 36, 40). Similar results were also found in an independent set of experiments (*SI Appendix*, Fig. S1 *D*–*F*). These data show that altered stomatal response kinetics to VPD can be analyzed with our system by direct comparisons to WT plants grown in parallel and analyzed within the same datasets (*Materials and Methods*).

**Fig. 1.**
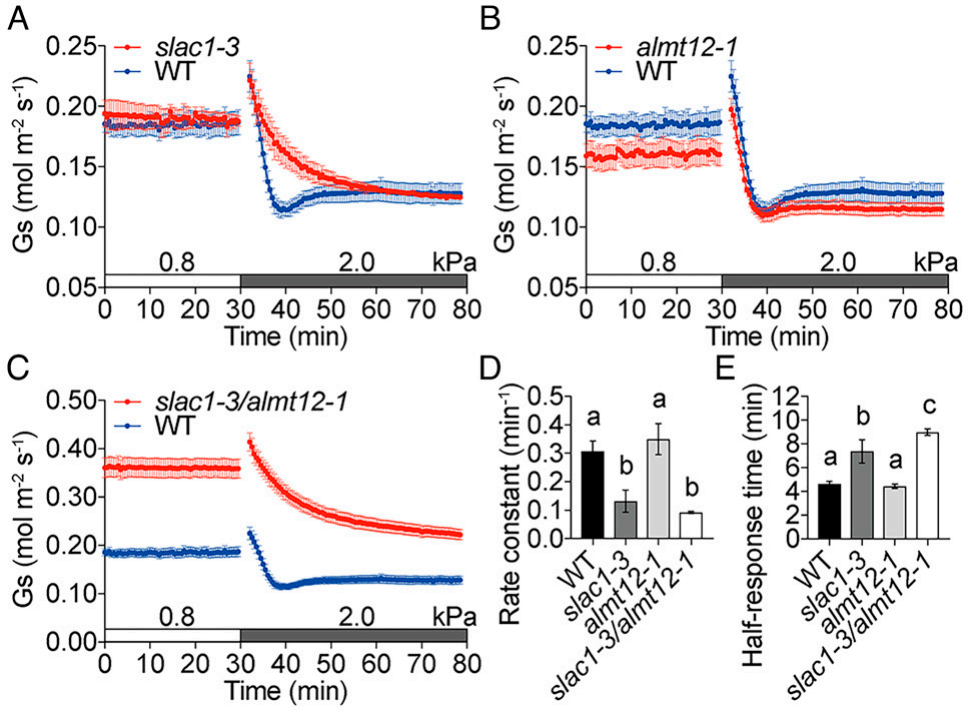
*slac1-3*/*almt12-1* double-mutant leaves have a higher stomatal conductance, whereas a delayed stomatal vapor pressure deficit response is associated with defective SLAC1 in *slac1-3* single and *slac1-3*/*almt12-1* double mutants. (*A*–*C*) Time-resolved stomatal conductance (*G*_s_) in response to increases in VPD in Col-0 (WT), *slac1-3*, *almt12-1*, and *slac1-3*/*almt12-1* mutant leaves under high light (450 μmol·m^−2^·s^−1^ red light combined with 50 μmol·m^−2^·s^−1^ blue light). VPDs are shown on top of the time axis in each panel. WT controls shown in *A*–*C* are the same because mutants and WT were grown in parallel and analyzed within the same experimental sets. (*D* and *E*) Average rate constants and average half-response times in response to VPD elevations in *A*–*C* and other figures are calculated and presented as described in *Materials and Methods*. Data represent mean ± SEM. Different letters represent significant differences (*P* < 0.05) analyzed by one-way ANOVA followed by Holm–Sidak multiple comparisons (*Results*). WT: *n* = 4 leaves; *slac1-3*: *n* = 3 leaves; and *almt12-1* and *slac1-3*/*almt12-1*: *n* = 5 leaves. Each analyzed leaf response was from a separate plant for all figures.

Activation of anion channels in guard cells plays an important role in stomatal closure. It has been shown that the slow-type anion channel SLAC1 is required for the high-VPD response (18). As a further test of our system, we analyzed the high-VPD response in *slac1-3* mutant leaves. Similar to the findings of Vahisalu et al. (18), *slac1-3* mutant leaves showed a slower response to high VPD (Fig. 1*A* and *SI Appendix*, Figs. S2 and 3). However, unlike *ost1-3* mutant leaves, the steady-state stomatal conductances at low-VPD and high-VPD conditions in *slac1-3* were comparable with WT under the imposed conditions (Fig. 1*A*). In another independent set of experiments, *slac1-3* mutant leaves exhibited slightly increased steady-state stomatal conductances compared to parallel-grown WT controls (*SI Appendix*, Fig. S3*A*).

It has been shown that the rapid-activating R-type anion channel, ALMT12/QUAC1, mediates anion efflux in guard cells during ABA-triggered, darkness-triggered, and high-CO_2_-triggered stomatal closure (15, 16, 55). To investigate whether ALMT12/QUAC1 is required for the high-VPD response, *altm12-1* mutant leaves were analyzed. Unexpectedly, no obvious difference in the high-VPD response was found between *altm12-1* mutant and WT leaves (Fig. 1*B*). We further analyzed the high-VPD response in *slac1-3*/*almt12-1* double mutants. Interestingly, *slac1-3*/*almt12-1* double-mutant leaves showed a higher steady-state stomatal conductance before and after high-VPD shifts compared to WT leaves and exhibited a slower stomatal closure in response to high-VPD stimulation, similar to *slac1-3* mutant leaves (Fig. 1*C* and *SI Appendix*, Fig. S3). Average rate constants in *slac1-3* and *slac1-3*/*almt12-1* mutant leaves were significantly smaller than in WT controls (Fig. 1*D*; *slac1-3*: 0.13 ± 0.04 min^−1^ vs. WT: 0.31 ± 0.04 min^−1^, *P* < 0.05 and *slac1-3*/*almt12-1*: 0.10 ± 0.00 min^−1^ vs. WT: 0.31 ± 0.04 min^−1^, *P* < 0.009 and *SI Appendix*, Fig. S3 *C* and *D*; *slac1-3*: 0.11 ± 0.01 min^−1^ vs. WT: 0.27 ± 0.03 min^−1^, *P* < 0.001 and *slac1-3*/*almt12-1*: 0.10 ± 0.00 min^−1^ vs. WT: 0.33 ± 0.03 min^−1^, *P* < 0.001), and average half-response times were larger in *slac1-3* and *slac1-3*/*almt12-1* mutant leaves than in WT controls (Fig. 1*E*; *slac1-3*: 7.4 ± 1.0 min vs. WT: 4.6 ± 0.2 min, *P* < 0.002 and *slac1-3*/*almt12-1*: 9.0 ± 0.3 min vs. WT: 4.6 ± 0.2 min, *P* < 0.001 and *SI Appendix*, Fig. S3 *E* and *F*; *slac1-3*: 8.4 ± 1.0 min vs. WT: 4.7 ± 0.2 min, *P* < 0.006 and *slac1-3*/*almt12-1*: 8.7 ± 0.3 min vs. WT: 4.8 ± 0.2 min, *P* < 0.001). These experiments provide further evidence that SLAC1 and AMLT1/QUAC1 synergistically function in controlling the steady-state stomatal conductance. Furthermore, the finding that stomatal closing occurs in *slac1-3*/*almt12-1* mutant leaves suggests that additional anion-channel genes could function in the VPD response.

### Investigation of Potential Roles of Calcium-Linked Signaling Mechanisms in High-VPD-Triggered Stomatal Closure.

**Fig. 2.**
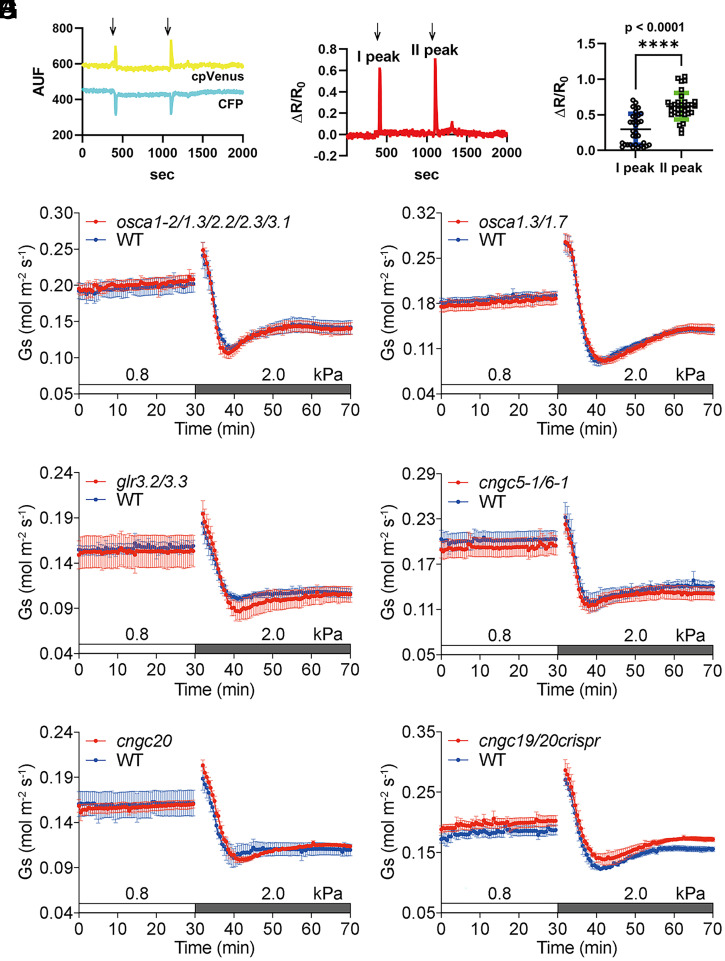
Both hyperosmotic and hypo-osmotic stress induce rapid cytoplasmic Ca^2+^ transients in guard cells, and no obvious stomatal VPD phenotype was found in the higher-order mutants of *OSCA*, *GLR3*, and *CNGC* Ca^2+^-permeable channels. (*A*) Representative single cpVenus and CFP emissions (540 and 480 nm) measured from a single guard cell of Col-0 (WT) *pGC1::YC3.60* plants initially bathed in a standard solution (5 mM KCl, 10 mM MES, and 50 μM Ca^2+^, pH 6.15) and then treated transiently with 500 mM sorbitol added to the same solution. Sorbitol was added and removed at the times indicated by arrows. (*B*) Representative normalized cpVenus/CFP ratio reported as Δ*R*/*R*_0_ calculated with the single emissions shown in *A*. (*C*) Scatter dot plot of maximal cytosolic Ca^2+^ increases in response to sorbitol addition (I peak) and removal (II peak) of *n* = 34 guard cells from six independent experiments. In blue and green are the mean and SD for the I and II peaks, respectively. AUF, arbitrary unit of fluorescence. ****P < 0.0001. (*D*–*I*) Time-resolved stomatal conductance (*G*_s_) responses to an increase in VPD in Col-0 (WT), *osca1-2*/*1.3*/*2.2*/*2.3*/*3*, *osca1.3*/*1.7*, *glr3.2*/*3.3*, *cngc5-1*/*6-1*, *cngc20*, and *cngc19*/*20crispr* mutant leaves under high light (450 μmol·m^−2^·s^−1^ red light combined with 50 μmol·m^−2^·s^−1^ blue light). VPDs are shown on top of the time axis in each panel. Data represent mean ± SEM. WT: *n* = 5 leaves in *D*–*F*, *n* = 4 leaves in *G* and *I*, and *n* = 3 leaves in *H*; *osca1-2*/*1.3*/*2.2*/*2.3*/*3*: *n* = 5 leaves; *osca1.3*/*1.7*: *n* = 5 leaves; *glr3.2*/*3.3*: *n* = 5 leaves; *cngc5-1*/*6-1*: *n* = 5 leaves; *cngc20*: *n* = 3 leaves; and *cngc19*/*20crispr*: *n* = 4 leaves.

It remains unclear how guard cells perceive and respond to increases in VPD to trigger stomatal closure. High VPD initially increases transpiration rates and leads to a rapid reduction of apoplastic water content in leaves (12). The alteration of water content and/or the initial transient increase in stomatal conductance, termed the wrong-way VPD response (56, 57) (e.g., Fig. 1), may trigger osmotic/mechanical-induced cytosolic calcium concentration changes in guard cells to trigger stomatal closure. We tested the effect of 500 mM sorbitol (as osmoticum) on the cytosolic Ca^2+^ concentration on preopened stomata in leaf epidermal strips of 4- to 5‐week‐old *Arabidopsis* plants expressing the Cameleon YC3.60 sensor placed under the control of the *pGC1* promoter (58). Both the addition of sorbitol (hyperosmotic stress) and removal of sorbitol (hypo‐osmotic stress) caused clear Förster resonance energy transfer (FRET) of the Cameleon sensor, with a decrease of cyan fluorescent protein (CFP) fluorescence and an increase in cpVenus fluorescence (Fig. 2*A*). When representing the data as normalized ratios (Δ*R*/*R*_0_), which depict cytosolic Ca^2+^ concentration changes, rapid and transient cytosolic [Ca^2+^] increases were observed after both the hyperosmotic and hypo-osmotic stimuli (Fig. 2*B*). Of note, the removal of sorbitol induced a higher maximum Ca^2+^ transient compared to the one observed after its addition (Fig. 2*C*).

Previous studies have indicated that OSCA1 and other OSCA family proteins are cation channels mediating calcium influx (59–64). Several studies have suggested that OSCA channels may be activated in response to osmotic or mechanical stimulation (59–63). We hypothesized that OSCA family proteins may be involved in transducing the high-VPD signal in guard cells. According to transcriptome datasets in the *Arabidopsis* Electronic Fluorescent Pictograph (eFP) browser, the transcripts of *OSCA1*, *OSCA1.3*, *OSCA2.2*, *OSCA2.3*, and *OSCA3.1* are generally more highly expressed in guard cells (*SI Appendix*, Fig. S4*A*) (58, 65). We therefore generated an *osca1-2*/*1.3*/*2.2*/*2.3*/*3.1* quintuple transfer DNA (T-DNA) insertion mutant, which included one reported intron allele in the *OSCA1* gene (59) and four exon alleles in the *OSCA1.3*, *2.2*, *2.3*, and *3.1* genes (*SI Appendix*, Fig. S5 *A* and *B*). However, the kinetics and steady-state stomatal conductance responses to VPD elevation in *osca1-2*/*1.3*/*2.2*/*2.3*/*3.1* quintuple mutant leaves did not show a clear difference compared to WT control leaves (Fig. 2*D*). Furthermore, we found that an *osca1.3*/*1.7* double-mutant allele, which is defective in elicitor-induced stomatal closure (66), exhibited WT-like high-VPD response (Fig. 2*E*).

To further investigate the potential roles of calcium-permeable channels in high-VPD-triggered stomatal closure, we focused on putative calcium channels with higher expression levels in guard-cell protoplasts, including glutamate-like receptors genes *GLR3.2* and *GLR3.3* and cyclic nucleotide-gated channel genes *CNGC5*, *CNGC6*, and *CNGC20* based on transcriptome analyses (*SI Appendix*, Fig. S4 *B*–*D*). Stomatal conductance responses to an increase in VPD were analyzed in *glr3.2*/*3.3*, *cngc5-1*/*6-1*, and *cngc20* T-DNA insertion mutants and a deletion mutant of two homologous tandem repeat genes *CNGC19* and *CNGC20*, *cngc19*/*20crispr* (Fig. 2 *F*–*I* and *SI Appendix*, Fig. S5 *C–F*). Notably, no obvious differences in the VPD responses were observed compared to parallel-grown WT leaves in all of these mutants.

The high-VPD response was also investigated in *cpk3*/*5*/*6*/*11*/*23* and *cpk3*/*4*/*5*/*6*/*11* quintuple mutants that include mutant alleles in several genes previously found to contribute to stomatal Ca^2+^ responses and/or anion-channel regulation (67–71). Furthermore, we investigated VPD responses in plasma-membrane-targeted Ca^2+^ sensor *cbl1*/*4*/*5*/*8*/*9* quintuple mutant leaves and in vacuolar Ca^2+^ sensor *cbl2*/*3rf* (reduced function) double-mutant leaves (71–74). No major differences in stomatal VPD change responses were found in these higher-order *cpk* quintuple and *cbl* mutants compared to WT leaves (*SI Appendix*, Fig. S6).

### High VPD and High CO_2_ Use Different Pathways to Trigger Stomatal Closure.

**Fig. 3.**
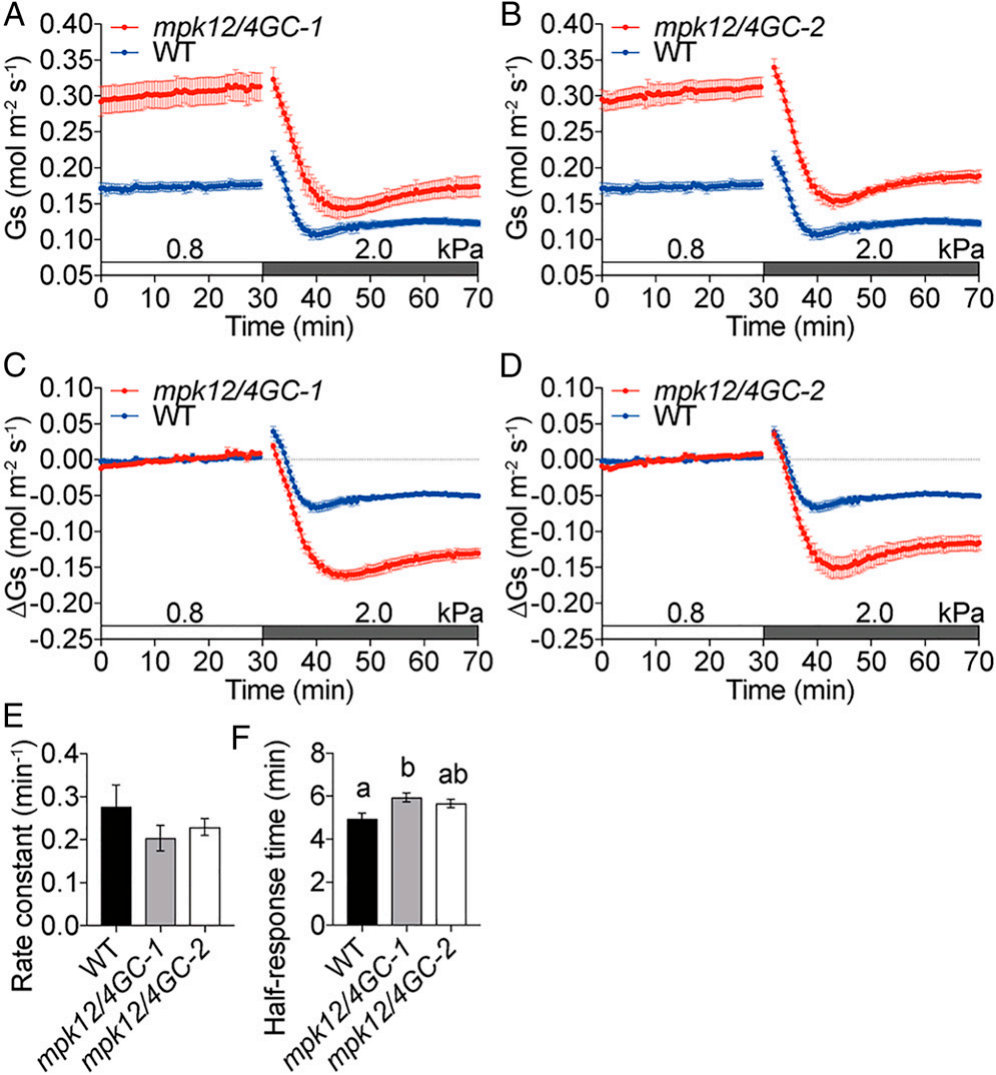
*mpk12*/*4GC* double mutants close stomata rapidly in response to VPD increase. (*A* and *B*) Time-resolved stomatal conductance (*G*_s_) in response to increases in VPD in Col-0 (WT), *mpk12*/*4GC-1*, and *mpk12*/*4GC-2* double-mutant leaves under high light (450 μmol·m^−2^·s^−1^ red light combined with 50 μmol·m^−2^·s^−1^ blue light). VPDs are shown on top of the time axis in each panel. Note that WT controls in *A*−*D* are the same, because mutants and WT were grown in parallel and analyzed within the same experimental sets. (*C* and *D*) Changes in stomatal conductances (Δ*G*_s_) were calculated as the difference between actual stomatal conductance and the average steady-state stomatal conductance at ∼0.8 kPa. (*E* and *F*) Average rate constants and average half-response times in response to VPD elevations are calculated and presented. Data represent mean ± SEM. Different letters represent significant differences (*P* < 0.05) analyzed by one-way ANOVA followed by Holm–Sidak multiple comparisons. *n* = 5 leaves for each genotype.

Defects in several ABA biosynthesis and ABA signaling mutants in *Arabidopsis* increase steady-state stomatal conductances and thus shift VPD responses to higher stomatal conductance values (5, 37). Recent time-resolved stomatal conductance analyses have led to the model that the rapid stomatal conductance response to VPD elevation occurs in parallel to the ABA signaling pathway (5, 34). A signaling pathway parallel to ABA signaling is the CO_2_ signaling pathway (46). MAP kinases MPK4 and MPK12 are essential for high-CO_2_-induced stomatal closure, but not for ABA-induced stomatal closure (75, 76). To investigate whether VPD and CO_2_ share a common signaling pathway upstream of anion-channel activation, the stomatal response to VPD elevation was analyzed in two independent *mpk12*/*4GC* double-mutant plant lines, in which guard cell *MPK4* was silenced by an artificial microRNA driven by the *MPK12* promoter in the *mpk12-4* mutant background (75, 77). These *mpk12*/*4GC* double-mutant alleles are the strongest presently known specific high-CO_2_-response impaired mutants in *Arabidopsis* (75). These analyses showed that the *mpk12*/*4GC* double-mutant leaves exhibited a much higher steady-state stomatal conductance than WT leaves (Fig. 3 *A* and *B*), consistent with previous findings (75). Previous research has shown a slightly lower stomatal index and no clear effect on stomatal density in *mpk12*/*4GC* double mutants (75). Leaves of *mpk12*/*4GC* double mutants responded rapidly to VPD elevations with response times similar to WT (Fig. 3 *A* and *B*). Relative changes in stomatal conductance at early time points in response to VPD increase (10 min) were even larger in these mutant leaves than in WT controls (Fig. 3 *C* and *D*). No difference in average rate constants was found between *mpk12*/*4GC* double mutants and WT (Fig. 3*E*; *mpk12*/*4GC-1*: 0.20 ± 0.03 min^−1^ vs. WT: 0.28 ± 0.05 min^−1^, *P* = 0.42; and *mpk12*/*4GC-2*: 0.23 ± 0.02 min^−1^ vs. WT: 0.28 ± 0.05 min^−1^, *P* = 0.58, tested by one-way ANOVA followed by Holm–Sidak multiple comparisons). Slightly, but not significantly, in one line, larger average half-response times in *mpk12*/*4GC* double mutants compared to WT controls were likely due to the large initial stomatal apertures (Fig. 3*F*; *mpk12*/*4GC-1*: 5.9 ± 0.2 min vs. WT: 5.0 ± 0.3 min, *P* < 0.03; and *mpk12*/*4GC-2*: 5.7 ± 0.2 min vs. WT: 5.0 ± 0.3 min, *P* = 0.08) (75). These findings that absolute high VPD–induced stomatal closing responses were stronger in *mpk12*/*4GC-2* double mutants than in WT controls provide evidence that high VPD and high CO_2_ trigger stomatal closure by different early signaling pathways.

### B3-Family Raf-Like Kinases Are Involved in High VPD–Induced Stomatal Closure.

**Fig. 4.**
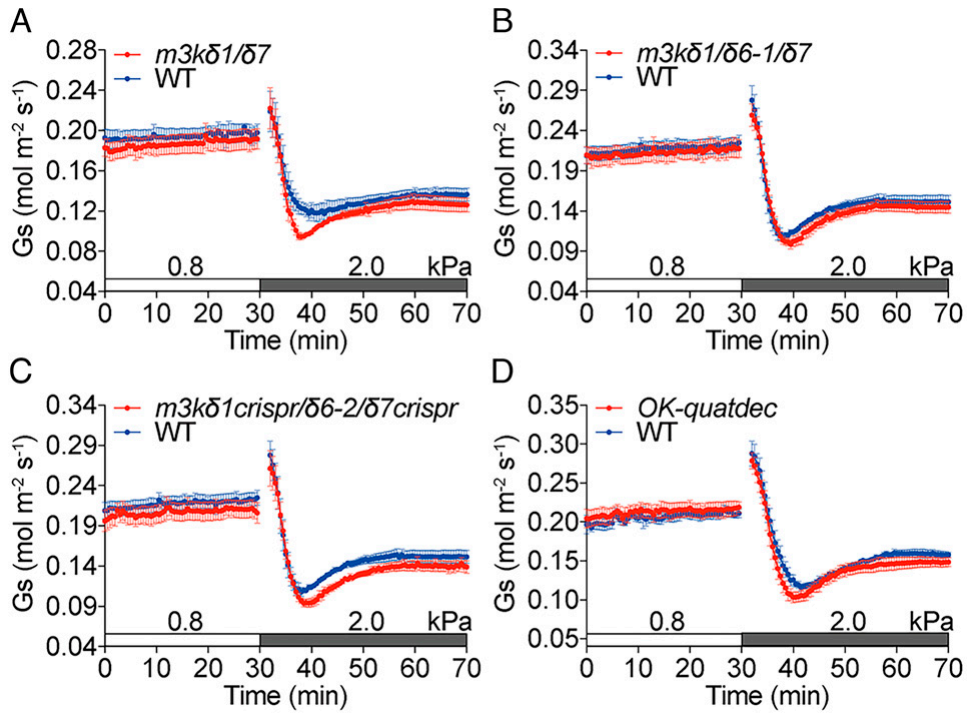
No obvious stomatal VPD phenotype is found in Raf-like kinase *m3kδ1*/δ7 double, *m3kδ1*/*δ6*/*δ7* triple, and *OK-quatdec* quattuordecuple mutants. (*A*–*D*) Time-resolved stomatal conductance (*G*_s_) in response to increases in VPD in Col-0 (WT), (*A*) Raf-like *m3kδ1*/*δ7* double mutant, (*B*) *m3kδ1*/*δ6-1*/*δ7* triple mutant, (*C*) *m3kδ1crispr*/*δ6-2*/*δ7crispr* triple mutant, and (*D*) *OK-quatdec* quattuordecuple mutant (*raf16*/*raf40*/*RAF24****^Δ10^***/*raf18*/*raf20*/*raf35*/*RAF42****^Δ6^***/*m3kδ1;raf3*/*m3kδ7;raf4*/*m3kδ6;raf5*/*raf7*/*raf8*/*raf9*/*raf10*) leaves under high light (450 μmol·m^−2^·s^−1^ red light combined with 50 μmol·m^−2^·s^−1^ blue light). Note that WT controls in *B* and *C* are the same because mutants and WT were grown in parallel and analyzed within the same experimental sets. Data represent mean ± SEM. *n* = 5 leaves from different plants in each genotype.

Recent studies identified B3-family Raf-like M3Ks as an important mechanism for the activation of SnRK2.2/SnRK2.3/OST1(SnRK2.6) protein kinases by ABA and osmotic stress (53, 54). Transcriptome datasets in the *Arabidopsis* eFP browser suggest that B3-family Raf-like M3Ks (*M3Kδ1*/*RAF3*, *M3Kδ3*/*EDR1*/*RAF2*, *M3Kδ5*/*RAF6*, *M3Kδ6*/*SIS8*/*RAF5*, *M3Kδ7*/*RAF4*, and *CTR1*/*RAF1*) are expressed in guard cells (*SI Appendix*, Fig. S4*D*). Our previous results showed that *m3kδ1*/*δ7* double, *m3kδ1*/*δ6-1*/*δ7* triple, and *m3kδ1crispr*/*δ6-2*/*δ7crispr* triple mutants exhibited partial degrees of ABA insensitivity in non-guard-cell tissues (54). Here, we examined whether M3Kδ1, M3Kδ6, and M3Kδ7 alone are required for the stomatal high-VPD response by analyzing these mutants. No obvious difference in stomatal VPD response was found between *m3kδ1*/*δ7* double, *m3kδ1*/*δ6-1*/*δ7* triple, and *m3kδ1crispr*/*δ6-2*/*δ7crispr* triple mutants compared to WT controls (Fig. 4 *A–C*). In addition to B3-family, B2- and B4-family Raf-like M3Ks are also involved in SnRK2 protein kinase activation in response to osmotic stress in plants (53, 78). We further investigated the stomatal high-VPD response in a B2-, B3-, and B4-family Raf-like kinase quattuordecuple mutant, *OK-quatdec* (*raf16*/*raf40*/*RAF24****^Δ10^***/*raf18*/*raf20*/*raf35*/*RAF42****^Δ6^***/*m3kδ1;raf3*/*m3kδ7;raf4*/*m3kδ6;raf5*/*raf7*/*raf8*/*raf9*/*raf10*), in which the mutations in 14 *Raf-like M3K kinase* genes were confirmed, as described in *Materials and Methods*. However, no obvious difference in the stomatal VPD response was found between *OK-quatdec* mutant plants compared to WT controls (Fig. 4*D*).

**Fig. 5.**
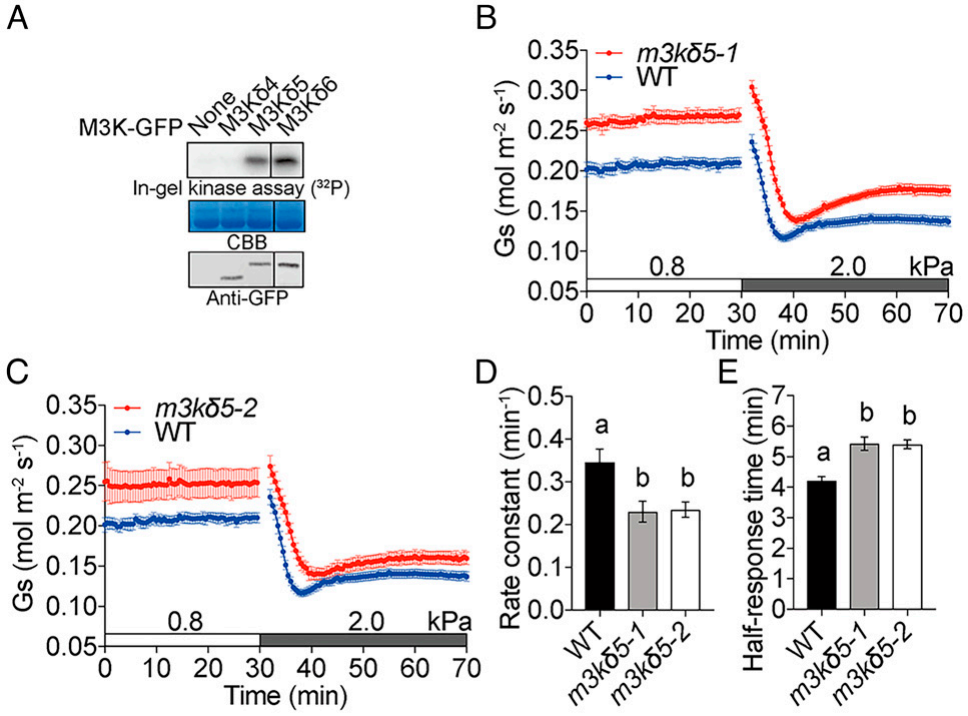
Stomatal VPD response is slightly slower in B3-family Raf-like kinase *m3kδ5* mutants. (*A*) The Raf-M3Kδ5 kinase domain activates OST1 in *Arabidopsis* mesophyll cells. The protein kinase domains of the Raf-like M3Ks were expressed transiently in mesophyll cell protoplasts isolated from *pUBQ10:OST1-HF Arabidopsis* leaves. OST1 kinase activity was visualized by in-gel kinase assays (*n* = 3). GFP, green fluorescent protein. CBB, coomassie brilliant blue loading control. Anti-GFP, immunoblotting of GFP-fusion proteins. (*B* and *C*) Time-resolved stomatal conductance (*G*_s_) in response to increases in VPD in Col-0 (WT) and Raf-like *m3kδ5* mutant under high light (450 μmol·m^−2^·s^−1^ red light combined with 50 μmol·m^−2^·s^−1^ blue light). WT controls shown in *B* and *C* are the same because mutants and WT were grown in parallel and analyzed within the same experimental sets. (*D* and *E*) Rate constants and half-response times in response to VPD elevations were calculated and presented. Data represent mean ± SEM. *n* = 5 for each genotype. Different letters represent significant differences (*P* < 0.05) analyzed by one-way ANOVA followed by Holm–Sidak multiple comparisons.

The above mutants did not include mutations in the *M3Kδ5* gene, which was the most highly expressed B3-family Raf-like *M3K* in guard cells (*SI Appendix*, Fig. S4*D*). We pursued in-gel kinase assays that showed that kinase domains of the B3-family member M3Kδ5 and M3Kδ6 kinase, but not the B2-family M3Kδ4 kinase, activated OST1/SnRK2.6 kinase in *Arabidopsis* mesophyll protoplasts (Fig. 5*A*). Stomatal VPD response assays showed that *m3kδ5-1* and *m3kδ5-2* single-mutant leaves exhibited increased steady-state stomatal conductances compared to WT controls (Fig. 5 *B* and *C*). Average rate constants in *m3kδ5-1* and *m3kδ5-2* single-mutant leaves were smaller than in WT controls (Fig. 5*D*; *m3kδ5-1*: 0.23 ± 0.02 min^−1^ vs. WT: 0.35 ± 0.03 min^−1^, *P* < 0.03; and *m3kδ5-2*: 0.23 ± 0.02 min^−1^ vs. WT: 0.35 ± 0.03 min^−1^, *P* < 0.03), and half-response times were larger in *m3kδ5-1* and *m3kδ5-2* mutant leaves than in WT controls (Fig. 5*E*; *m3kδ5-1*: 5.4 ± 0.2 min vs. WT: 4.2 ± 0.1 min, *P* < 0.001; and *m3kδ5-2*: 5.4 ± 0.1 min vs. WT: 4.2 ± 0.1 min, *P* < 0.001).

**Fig. 6.**
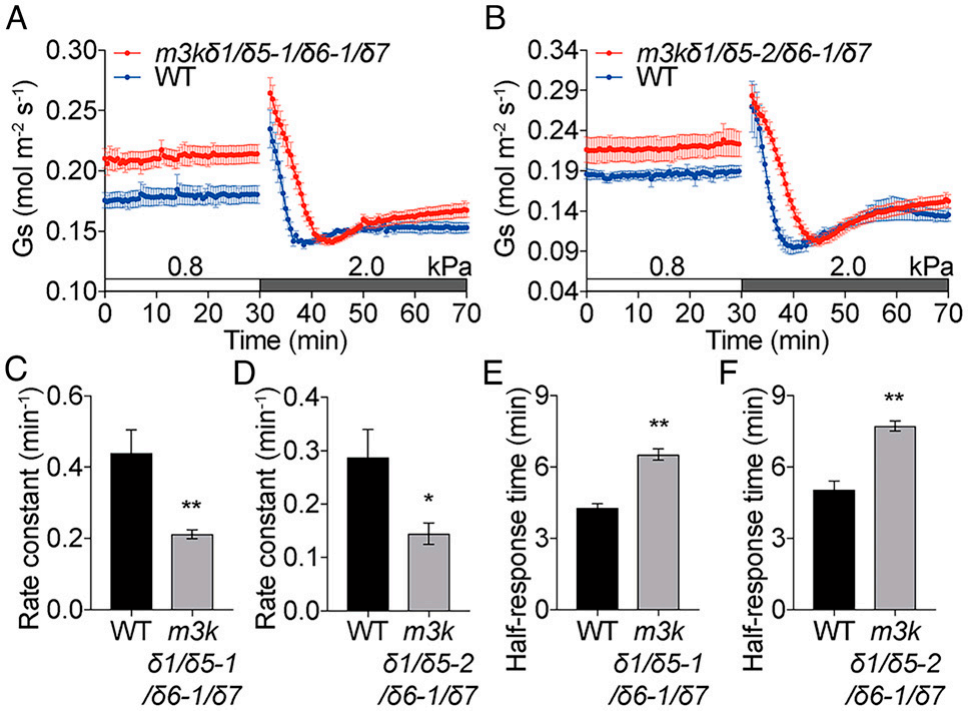
Stomatal VPD response is slower in B3-family Raf-like kinase *m3kδ1*/*δ5*/*δ6*/δ7 quadruple mutants. (*A* and *B*) Time-resolved stomatal conductance (*G*_s_) in response to increases in VPD in Col-0 (WT), Raf-like *m3kδ1*/*δ5-1*/*δ6-1*/*δ7* quadruple-mutant, and *m3kδ1*/*δ5-2*/*δ6-1*/*δ7* quadruple-mutant leaves under high light (450 μmol·m^−2^·s^−1^ red light combined with 50 μmol·m^−2^·s^−1^ blue light). VPDs are shown on top of the time axis in each panel. (*C*–*F*) Average rate constants (*C* and *D*) and average half-response times (*E* and *F*) in response to VPD elevations were calculated and presented. Data represent mean ± SEM. *n* = 5 for each genotype in *A*. *n* = 3 for WT and *n* = 4 for the *m3kδ1*/*δ5-2*/*δ6-1*/*δ7* quadruple mutant in *B*. *P < 0.05; **P < 0.01 (analyzed by Student’s *t* test compared to WT control). Data represent one of two independent sets of experiments in each mutant allele showing similar results.

Based on findings with the two *m3kδ5* mutant alleles, *m3kδ1*/*δ5-1*/*δ6-1*/*δ7* and *m3kδ1*/*δ5-2*/*δ6-1*/*δ7* quadruple mutants were generated to investigate their VPD responses. Both mutant alleles exhibited larger steady-state stomatal conductances under low-VPD control conditions in both high-light (Fig. 6; 500 μmol⋅m^−2^⋅s^−1^) and low-light (*SI Appendix*, Fig. S7; 150 μmol⋅m^−2^⋅s^−1^) conditions compared to WT controls (Fig. 6 *A* and *B* and *SI Appendix*, Fig. S7 *A* and *B*). Furthermore, average rate constants of high VPD–induced stomatal closing in *m3kδ1*/*δ5-1*/*δ6-1*/*δ7* and *m3kδ1*/*δ5-2*/*δ6-1*/*δ7* quadruple-mutant leaves were reproducibly and significantly smaller than in the WT controls grown in parallel (Fig. 6 *C* and *D*; *m3kδ1*/*δ5-1*/*δ6-1*/*δ7*: 0.21 ± 0.01 min^−1^ vs. WT: 0.44 ± 0.06 min^−1^, *P* < 0.009 and *m3kδ1*/*δ5-2*/*δ6-1*/*δ7*: 0.14 ± 0.02 min^−1^ vs. WT: 0.29 ± 0.05 min^−1^, *P* < 0.04 and *SI Appendix*, Fig. S7*C*; *m3kδ1*/*δ5-1*/*δ6-1*/*δ7*: 0.11 ± 0.03 min^−1^ vs. WT: 0.40 ± 0.05 min^−1^, *P* < 0.001 and *m3kδ1*/*δ5-2*/*δ6-1*/*δ7*: 0.13 ± 0.2 min^−1^ vs. WT: 0.40 ± 0.05 min^−1^, *P* < 0.001). Half-response times were significantly larger in *m3kδ1*/*δ5-1*/*δ6-1*/*δ7* and *m3kδ1*/*δ5-2*/*δ6-1*/*δ7* quadruple-mutant leaves than in the WT controls at both light intensities (Fig. 6 *E* and *F*; *m3kδ1*/*δ5-1*/*δ6-1*/*δ7*: 6.5 ± 0.2 min vs. WT: 4.3 ± 0.2 min, *P* < 0.001 and *m3kδ1*/*δ5-2*/*δ6-1*/*δ7*: 7.7 ± 0.2 min vs. WT: 5.0 ± 0.4 min, *P* < 0.002 and *SI Appendix*, Fig. S7*D*; *m3kδ1*/*δ5-1*/*δ6-1*/*δ7*: 8.3 ± 0.5 min vs. WT: 4.4 ± 0.3 min, *P* < 0.001 and *m3kδ1*/*δ5-2*/*δ6-1*/*δ7*: 7.9 ± 0.4 min vs. WT: 4.4 ± 0.3 min, *P* < 0.001). These data at high (Fig. 6) and low (*SI Appendix*, Fig. S7) light intensities provide evidence that the phenotypes found in the *m3kδ1*/*δ5*/*δ6*/*δ7* quadruple-mutant alleles are not simply a result of the higher steady-state conductance in these mutants compared to WT plants.

As the slowing of the VPD response in these quadruple-mutant alleles was significantly, but only slightly, slower, we investigated these mutants in the independent laboratory of E.M. in genotype-blinded experiments using a distinct whole-plant gas-exchange system. Higher stomatal conductances and in part increased half-response times in *m3kδ1*/*δ5*/*δ6*/*δ7* quadruple mutants were independently found in intact whole-plant gas-exchange analyses (*SI Appendix*, Fig. S8). These results suggest that B3-family Raf-like M3Ks play a role in stomatal closure induced by VPD elevation.

### GHR1 Is Required for High VPD–Induced Stomatal Closure.

**Fig. 7.**
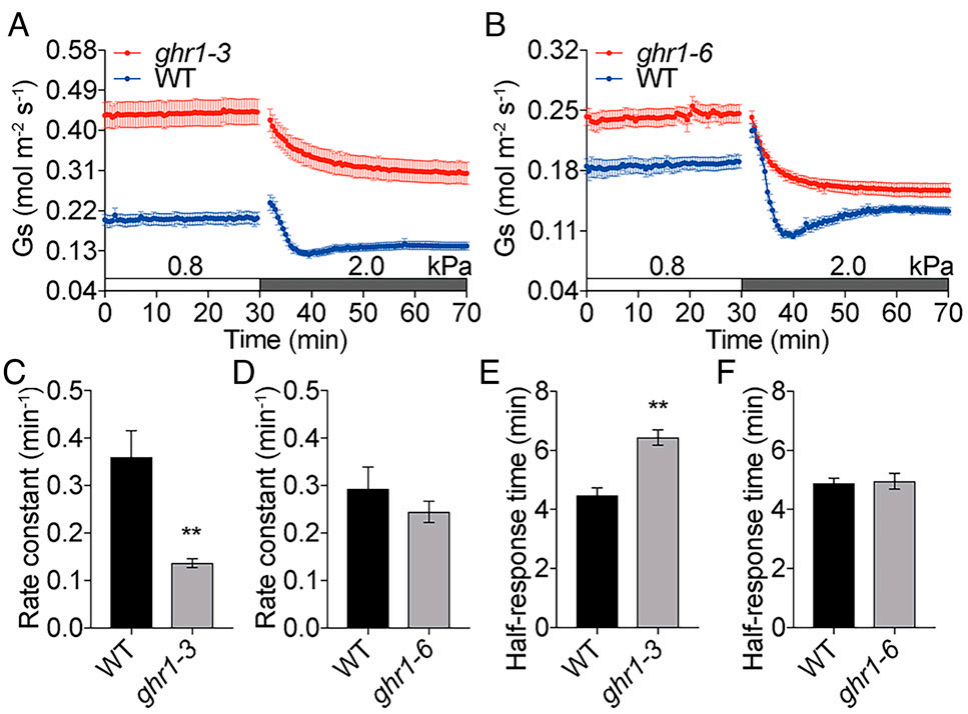
Stomatal VPD responses, including the initial transient increase in stomatal conductance, are impaired in *ghr1* mutants. (*A* and *B*) Time-resolved stomatal conductance (*G*_s_) in response to increases in VPD in Col-0 (WT) and *ghr1-3*, and *ghr1-6* mutant leaves under high light (450 μmol·m^−2^·s^−1^ red light combined with 50 μmol·m^−2^·s^−1^ blue light). VPDs are shown on top of the time axis in each panel. (*C*–*F*) Average rate constants (*C* and *D*) and average half-response times (*E* and *F*) in response to VPD elevations were calculated and presented as described. Data represent mean ± SEM. **P < 0.01 (as analyzed by Student’s *t* test compared to WT control). WT: *n* = 4 leaves in *A* and *n* = 5 leaves in *B*; *ghr1-3*: *n* = 5 leaves; and *ghr1-6*: *n* = 5 leaves. Data represent one of two independent sets of experiments. Similar results were found in an independent set of experiments in each mutant allele (*SI Appendix*, Fig. S8).

*GHR1* encodes a receptor-like (pseudo)kinase, which is involved in activating the S-type anion channel SLAC1 to trigger stomatal closure (77, 79, 80). The involvement of GHR1 in the stomatal high-VPD response was analyzed in *ghr1-3* and *ghr1-6* exon-insertion mutant leaves. Leaves of the strong *ghr1-3* allele and the weak *ghr1-6* allele mutants (80) showed higher steady-state stomatal conductances in both high-VPD and low-VPD conditions compared to WT controls (Fig. 7 *A* and *B* and *SI Appendix*, Fig. S9 *A* and *B*). Although high-VPD treatment triggered a degree of stomatal closure in *ghr1-3* and *ghr1-6* mutant leaves, *ghr1* mutant leaves exhibited a clear difference in response kinetics compared to WT leaves under both high-light (500 μmol⋅m^−2^⋅s^−1^; Fig. 7 *A* and *B* and *SI Appendix*, Fig. S9 *A* and *B*) and low-light (150 μmol⋅m^−2^⋅s^−1^; *SI Appendix*, Fig. S9*C*) experimental conditions. Rate constants in the strong *ghr1-3* mutant leaves were significantly smaller than in WT leaves (Fig. 7 *C* and *D*; *ghr1-3*: 0.14 ± 0.01 min^−1^ vs. WT: 0.36 ± 0.06 min^−1^, *P* < 0.003 and *ghr1-6*: 0.24 ± 0.02 min^−1^ vs. WT: 0.29 ± 0.05 min^−1^, *P* = 0.37 and *SI Appendix*, Fig. S9 *D*–*F*; *ghr1-3*: 0.15 ± 0.00 min^−1^ vs. WT: 0.44 ± 0.07 min^−1^, *P* < 0.003; *ghr1-6*: 0.25 ± 0.02 min^−1^ vs. WT: 0.42 ± 0.04 min^−1^, *P* < 0.03; *ghr1-3*: 0.15 ± 0.01 min^−1^ vs. WT: 0.41 ± 0.08 min^−1^, *P* < 0.03; and *ghr1-6*: 0.26 ± 0.05 min^−1^ vs. WT: 0.41 ± 0.08 min^−1^, *P* = 0.15). Half-response times were larger in strong *ghr1-3* mutant leaves than in WT controls (Fig. 7 *E* and *F*; *ghr1-3*: 6.4 ± 0.3 min vs. WT: 4.5 ± 0.2 min, *P* < 0.002 and *ghr1-6*: 5.0 ± 0.3 min vs. WT: 4.9 ± 0.2 min, *P* = 0.83 and *SI Appendix*, Fig. S9 *G*–*I*; *ghr1-3*: 4.1 ± 0.3 min vs. WT: 6.3 ± 0.1 min, *P* < 0.001; *ghr1-6*: 4.6 ± 0.3 min vs. WT: 4.2 ± 0.2 min, *P* = 0.33; *ghr1-3*: 5.9 ± 0.1 min vs. WT: 4.2 ± 0.5 min, *P* < 0.03; and *ghr1-6*: 5.0 ± 0.3 min vs. WT: 4.2 ± 0.5 min, *P* = 0.17). Taken together, our results show that GHR1 is required for the rapid stomatal closure induced in response to VPD increases. Furthermore, in contrast to all other mutants investigated in the present study, *ghr1* mutants showed no initial transient stomatal conductance increase (wrong-way response) as found under high-light conditions (Fig. 7 *A* and *B*).

## Discussion

The continuing elevation in global temperature is suggested to lead to a trend of rising vapor pressure deficit, though VPD is variable depending on conditions (81). Increases in VPD affect transpiration and regulate stomatal conductance of terrestrial plants (82–85). The molecular mechanisms of VPD sensing and signal transduction remain to a large degree unknown. Previous studies have focused on the roles of ABA in the VPD response (introduction). In the present study, we have investigated mutants affected in several stomatal responses. Mutants that disrupt specific known signaling mechanisms or pathways were investigated.

We show here that *mpk12*/*mpk4GC* mutant alleles that disrupt early stomatal CO_2_ signal transduction (75) show robust stomatal closing responses to VPD elevation. Osmotic stress exposures caused rapid cytoplasmic Ca^2+^ transients in guard cells (Fig. 2 *A–C*). Several higher-order calcium-permeable channel mutants (*osca1-2*/*1.3*/*2.2*/*2.3*/*3.1* quintuple, *osca1.3*/*1.7* double, *glr3.2*/*3.3* double, *cngc5-1*/*6-1* double, *cngc20* single, and *cngc19*/*20crispr* double) and calcium-signaling mutants (*cpk3*/*5*/*6*/*11*/*23* quintuple, *cpk3*/*4*/*5*/*6*/*11* quintuple, *cbl1*/*4*/*5*/*8*/*9* quintuple, and *cbl2*/*3rf* double) were analyzed, but were not affected in their high-VPD-triggered stomatal closing responses. Interestingly, however, we uncover here that the recently identified B3 family members of M3K RAF-like kinases, particularly M3Kδ5, but not B4 RAF-like M3Ks, are required for an intact rapid stomatal VPD response. Furthermore, we reveal that the transmembrane receptor-like pseudokinase GHR1 functions in the stomatal response to VPD elevation.

Analyses of the *ost1-3* single mutant (*SI Appendix*, Fig. S1) confirmed that OST1/SnRK2.6 is a central regulator of hydroactive high VPD–induced stomatal closure (5, 36, 39) and provided an internal control for our analysis system. The OST1 protein kinase regulates both S-type anion channels and the R-type anion channel ALMT12/QUAC1 in guard cells or *Xenopus* oocytes in response to several stimuli (28, 86–88). In the present study, analyses of *slac1-3* single-mutant leaves were used as an additional system control, confirming that the S-type anion channel SLAC1 contributes to high-VPD-mediated stomatal closing (Fig. 1 *A*, *D*, and *E* and *SI Appendix*, Fig. S3 *A*, *C*, and *D*) (18).

Notably, the single *almt12-1* mutant exhibited high VPD–induced stomatal closing kinetics similar to WT plants (Fig. 1 *B*, *D*, and *E*). Interestingly, however, *slac1-3*/*almt12-1* double-mutant leaves showed a dramatic increase in the steady-state stomatal conductance and a slowing of the high-VPD response, which was kinetically similar to the *slac1-3* single mutant (Fig. 1 *C*, *D*, and *E* and *SI Appendix*, Fig. S3). These results indicate that S-type anion channels play a rate-limiting role, and R-type anion channels function together with S-type anion channels in setting the steady-state stomatal conductance under the imposed conditions. Given redundancies and nonlinearities in signal-transduction networks (89, 90), our data do not unequivocally exclude a contribution of R-type anion channels to the high VPD–induced stomatal closing response. The slow, but continued, stomatal closing response in *slac1*/*almt12* double-mutant leaves suggests that additional anion channels and mechanisms function in parallel to these channels.

In this study, higher steady-state stomatal conductances in *m3k*δ5 single-mutant and *m3kδ1*/*δ5*/*δ6*/*δ7* quadruple-mutant alleles were not attributable to increases in stomatal index (*SI Appendix*, Fig. S10). Furthermore, the slower response to an increase in VPD in *m3kδ1*/*δ5*/*δ6*/*δ7* quadruple mutants was not simply caused by the increased initial steady-state stomatal conductance in these quadruple mutants for several reasons. First, we found that the larger half-response time in *m3kδ1*/*δ5*/*δ6*/*δ7* quadruple mutants was consistently observed, even at low light, with reduced initial steady-state stomatal conductance levels (*SI Appendix*, Fig. S7). Second, unlike *m3kδ1*/*δ5*/*δ6*/*δ7* quadruple-mutant alleles, no significant differences in half-response times were found in additional controls in WT plants exposed to high-light and low-light conditions under 400 parts per million (ppm) CO_2_ or 150 ppm CO_2_ (*SI Appendix*, Fig. S11). Third, a rapid stomatal response to increases in VPD was observed in the early CO_2_ signaling *mpk12*/*4GC* mutant lines that also exhibited high initial steady-state stomatal conductance (Fig. 3). Therefore, our findings suggest that B3 M3Ks are involved in accelerating the hydroactive signaling response to an increase in VPD.

Previous studies showed that ABA and hyperosmolarity activate the OST1/SnRK2.6/SRK2E protein kinase in *Arabidopsis* culture cells, roots, and seedlings (32, 50, 91, 92). Moreover, the OST1/SnRK2.6/SRK2E-like protein kinases are also activated by ABA in guard cells (31, 47, 93–96). Although low humidity/high VPD activates the OST1/SnRK2.6/SRK2E protein kinase in leaves (50), it remains to be directly determined whether, and if so how, low humidity may enhance OST1/SnRK2s activity in guard cells. In-gel kinase assays demonstrated that Raf-like kinases are required for OST1/SnRK2s kinase activation during ABA and hyperosmolarity stimulation in seedlings (52–54, 78). In the present study, we have found that the guard-cell-expressed M3Kδ5 protein kinase phosphorylates and activates the OST1/SnRK2.6 in plants (Fig. 5*A*). The higher basal stomatal conductance in *m3kδ5* single-mutant alleles and *m3kδ1*/*δ5*/*δ6*/*δ7* quadruple mutants (Fig. 5 and 6 and *SI Appendix*, Fig. S7) may be due to a lower basal OST1 activity in mutant guard cells. The slower high VPD–induced stomatal closure in *m3kδ1*/*δ5*/*δ6*/*δ7* quadruple-mutant leaves (Fig. 6 and *SI Appendix*, Fig. S7) may be caused by a reduction in Raf-like kinase-mediated OST1/SnRK2s activation in guard cells. Time-resolved analyses of OST1/SnRK2 protein kinase activity (47) in response to VPD changes could allow testing of this model.

Unlike the *m3kδ5* single-mutant and *m3kδ1*/*δ5*/*δ6*/*δ7* quadruple-mutant alleles, no obvious high-VPD-triggered stomatal response phenotype was observed in *m3kδ1*/*δ7* double, *m3kδ1*/*δ6*/*δ7* triple, and in an *OK-quatdec* quattuordecuple mutant (Fig. 4*D*). There are 80 *M3K* genes encoded in the *Arabidopsis* genome (97–99). The *OK-quatdec* line includes mutations in 14 Raf-like kinase genes, including several osmotic stress-activated B4 family members (53). The present findings indicate a less important role of these B4-family Raf-kinase members in the VPD response, compared to the osmotic stress response in whole seedlings. The present results further suggest that M3Kδ5/RAF6 plays an important role in VPD-induced stomatal regulation.

A previous study suggested that the receptor-like pseudokinase GHR1 mediates stomatal closure in response to ABA, CO_2_, ozone, and darkness, but may not be required for high-VPD-triggered stomatal closure (80). In the present study, the severe *ghr1-3* mutant allele showed a clear impairment in high VPD–induced stomatal closure (Fig. 7 and *SI Appendix*, Fig. S9). GHR1 can directly activate the SLAC1 anion channel in *Xenopus* oocytes when these two proteins are artificially linked via BiFC constructs (79, 80). GHR1 is required for stomatal closure in response to ABA, hydrogen peroxide, ozone, high CO_2_, and low light intensity (79, 80). GHR1-mediated activation of the S-type anion channel SLAC1 is negatively regulated by the protein phosphatase 2C, ABI2, in *Xenopus* oocytes (77, 79). As we show that early CO_2_ signaling is not required for the stomatal high-VPD response (Fig. 3), the defect in the stomatal high-VPD response in *ghr1* mutant leaves is likely caused by a GHR1 function downstream of early CO_2_ signaling or a different signaling pathway that makes use of GHR1. It has been suggested that the receptor-like pseudokinase GHR1 serves as a scaffolding protein to recruit the calcium-dependent protein kinase CPK3 for regulating SLAC1 anion-channel activation (80). Higher-order *cpk* mutants analyzed here included *cpk3* mutation, but did not affect the high-VPD response (*SI Appendix*, Fig. S6 *A* and *B*). Therefore, GHR1 may recruit additional, yet to be discovered signaling components to regulate the stomatal high-VPD response.

Classical research showed that high VPD/low humidity triggers a transient stomatal opening before stomatal closing (56, 100–102). Notably, this transient increase in stomatal conductance in response to an increase in VPD (wrong-way response) may in part result from a simplified assumption of saturated vapor pressure inside leaves during stomatal conductance recordings at high stomatal conductances (103). The wrong-way response was found in WT and most of the studied mutants, including *ost1-3*, *slac1-3*, *slac1-3*/*almt12-1*, and *mpk12*/*4GC* mutants, with high steady-state stomatal conductances. These data indicate that the larger stomatal conductance of *ghr1* leaves is not solely due to the above-simplified assumption. Interestingly, the lack of a clear wrong-way response observed in both *ghr1* mutant alleles points to an additional role of GHR1. It has been proposed that the initial hydropassive loss of turgor in epidermal cells adjacent to guard cells during high-VPD/low-humidity stimulation drives the transient stomatal opening (2, 104). In a previous study, a transient stomatal opening could only be observed in stomata with living subsidiary cells in *Tradescantia pallida* or when guard cells were surrounded by living epidermal cells in *Vicia faba* (3). Therefore, the lack of high-VPD-triggered transient stomatal opening in *ghr1* mutants suggests a function of GHR1 in the mechanical interaction between guard cells and neighboring pavement cells.

It remains to be elucidated whether mechanical and hyperosmolarity-sensing mechanisms are involved in high VPD–induced stomatal closure. OSCA channels have been reported to be mechanical- and osmotic-sensing calcium channels in higher plants, with structural analyses resolving possible mechanisms (59–64). In the present study, we generated higher-order *osca* mutants, based on guard-cell transcriptome analyses from independent laboratories (*SI Appendix*, Fig. S4) (58, 65). However, the stomatal responses to high VPD were not altered in *osca1-2*/*1.3*/*2.2*/*2.3*/*3.1* quintuple-mutant leaves (Fig. 2*D*), and hyperosmotic stress was insufficient to activate the rice ortholog OsOSCA2;1 when expressed alone in *Xenopus* oocytes (64). It has been reported that the activation of Raf-like kinases or SnRK2s by hyperosmolarity was not altered in an *osca* septuple mutant (53). These results suggest that high VPD–induced stomatal closure may be independent of OSCAs and, in particular, the five more guard-cell-expressed OSCAs analyzed here. A recent study has identified an additional OSCA that is not highly expressed in guard cells, OSCA1.7, but is together with OSCA1.3 required for PAMP-induced stomatal closing during pathogen signaling (66). However, we found WT-like high-VPD responses in *osca1.3*/*1.7* double-mutant leaves. We show that guard-cell cytosolic Ca^2+^ concentrations transiently increase in response to both hyperosmolality and hypo-osmolality (Fig. 2 *A–C*), but whether Ca^2+^ signaling is required for the stomatal high-VPD response remains to be investigated. Although no obvious stomatal closure phenotype in response to VPD elevation was found in the investigated calcium-permeable channel mutants, *cpk* quintuple, and higher-order *cbl* mutants (Fig. 2 *D*–*G* and *SI Appendix*, Fig. S6), we do not exclude a possible role of calcium in stomatal high-VPD signal transduction. Additional higher-order mutants in Ca^2+^ signaling components may be needed to impair the stomatal high-VPD response.

In summary, the identification of B3-family Raf-like kinases and the transmembrane receptor-like GHR1 in the stomatal VPD response advances the understanding of molecular and genetic mechanisms that mediate the hydroactive stomatal VPD response. Interestingly, the initial transient stomatal opening wrong-way response to a VPD increase that precedes stomatal closing (56, 100) was disrupted in *ghr1* mutant alleles, indicating a function of GHR1 in guard-cell–pavement-cell communication during VPD signaling. Furthermore, our data exclude the involvement of the early CO_2_ signal-transduction pathway in rapid high VPD–induced stomatal closure. Further research will be required to elucidate the upstream high VPD–sensing mechanisms, which are shown here to require B3-family Raf-like kinases and the receptor-like (pseudo) protein kinase GHR1. Understanding the detailed molecular and genetic mechanisms underlying the VPD response is relevant for models that predict plant transpiration in response to climate change and could become relevant for the future development of plants with enhanced resilience to climate change.

## Materials and Methods

### Plant Materials and Growth Conditions.

*Arabidopsis thaliana* Columbia-0 (Col-0) ecotype plants were used as WT. *Arabidopsis* mutant lines investigated in this study were: *ost1-3* (32), *slac1-3* (17, 18), *almt12-1* (16), *slac1-3*/*almt12-1*, *osca1.3*/*1.7* (66), *glr3.2*/*3.3* (105), *cngc5-1*/*6-1* (106), *cngc20* (Salk_129133), *cpk3*/*5*/*6*/*11*/*23*, *cpk3*/*4*/*5*/*6*/*11* (71), *cbl1*/*4*/*5*/*8*/*9* (74), *cbl2*/*3rf* (72), *mpk12*/*4GC* (75), *m3kδ1*/*δ7*, *m3kδ1*/*δ6-1*/*δ7*, *m3kδ1crispr*/*δ6-2*/*δ7crispr* (54), *OK-quatdec* (53), *ghr1-3*, and *ghr1-6* (80). An *osca1-2*/*1.3*/*2.2*/*2.3*/*3.1* quintuple mutant was generated from *osca1-2* (59), *osca1.3* (SALK_129246), *osca2.2* (SALK_131951), *osca2.3* (SALK_141893), and *osca3.1* (SALK_078537) by genetic crossing. A *cngc19*/*20crispr* deletion double mutant was generated by the CRISPR/Cas9 technique, as described in *SI Appendix*, Fig. S5*F*. *m3kδ1*/*δ5-1*/*δ6-1*/*δ7* and *m3kδ1*/*δ5-2*/*δ6-1*/*δ7* quadruple mutants were generated by crossing *m3kδ5-1* (Salk_025685) or *m3kδ5-2* (Salk_149401) with *m3kδ1*/*δ6-1*/*δ7*. Primers used for genotyping are listed in *SI Appendix*, Table S1. Plants were grown in soil in Percival growth cabinets at a 12-/12-h, 21/21 °C day/night cycle, a photosynthetic photon flux density of 90 to 110 μmol·m^−2^·s^−1^, and 70 to 80% relative humidity. Plants were watered two times per week to prevent soil drying and drought stress.

### Time-Resolved Intact VPD-Dependent Leaf Stomatal Conductance Experiments.

Stomatal conductance recordings from intact leaf 8 to leaf 12 of 5.5- to 8-wk-old plants in different batches of experiments were conducted starting 1 to 2 h after growth-chamber light onset. An LI-6800 Portable Photosynthesis System with an integrated Multiphase Flash Fluorometer (6800-01A; Li-Cor Inc.) was used to measure gas exchange in *Arabidopsis* leaves. Leaves were clamped and kept at 400 ppm ambient CO_2_; 21 °C heat-exchanger temperature; 0.8 kPa VPD; 450 μmol·m^−2^·s^−1^ red light combined with 50 μmol·m^−2^·s^−1^ blue light in most of the experiments, as done in other studies analyzing the VPD response (5); and 500 μmol·s^−1^ incoming airflow rate for 1.5 to 2.5 h until stomatal conductance stabilized. Some experiments presented in *SI Appendix* were performed under lower light intensity at 135 μmol·m^−2^·s^−1^ red light combined with 15 μmol·m^−2^·s^−1^ blue light, as described in the figure legends. For stomatal responses to VPD shifts, stomatal conductances were recorded every 30 s at ∼0.8 kPa VPD (65 to 72% relative air humidity), followed by ∼2.0 kPa VPD (20 to 32% relative air humidity) in most experiments, as indicated in the figures. VPD values were computed internally by the gas-exchange system. Stomatal conductance measurements in response to VPD shifts are affected by multiple parameters and therefore should be viewed as semiquantitative relative responses. Therefore, parallel-grown WT control plants were investigated in all experiments, which enabled direct resolution of non-WT-like impaired responses in mutants. Furthermore, the following measures were taken in these experiments. Matching between sample and reference infrared gas analyzers was executed before each experiment and 50 min after shifting to high VPD to adjust the errors due to the changes in humidity. The stomatal conductances at high VPD in each experiment were corrected by the differences in average stomatal conductances between 5 min before matching and 3 to 8 min after matching. The values of stomatal conductances were plotted from 2 min to 40 min or 50 min after VPD increase as in the figures. The first 2-min data points were omitted because of the inaccurate read and calculation of stomatal conductances before VPD reached more reliable values. High-VPD response rate constants were approximated by exponential one-phase decays starting from 2 min after shifting to high VPD to time points that that showed the minimum stomatal conductances in the investigated genotypes and in independent experiments using GraphPad Prism 7.0e software. Stomatal conductances at the following time points after high-VPD shifts were analyzed during curve fitting as follows: 2 to ∼10 min: WT in Fig. 5*D*; 2 to ∼12 min: WT in Figs. 1*D*, 3*E*, 6*C*, and 7 *C* and *D* and *SI Appendix*, Figs. S3*C* and S9 *D* and *E* and *almt12-1* in Fig. 1*D*; 2 to ∼14 min: WT in Fig. 6D and *SI Appendix*, Figs. S1*B*, S3*D*, S7*C*, and S9*F* and *m3kδ5* mutant alleles in Fig. 5*D*; 2 to ∼18 min: *mpk12*/*4GC* mutant alleles in Fig. 3E; 2 to ∼20 min: *m3kδ1*/*δ5*/*δ6*/*δ7* mutant alleles in Fig. 6 *C* and *D* and *SI Appendix*, Fig. S7*C*; 2 min to the end of the experiment: WT in *SI Appendix*, Fig. S1*E*, *ost1-3* in *SI Appendix*, Fig. S1 *B* and *E*, *slac1-3* in Fig. 1*D* and *SI Appendix*, Fig. S3*C*;,*slac1-3*/*almt12-1* in Fig. 1*D* and *SI Appendix*, Fig. S3*D*, and *ghr1* mutant alleles in Fig. 7 *C* and *D* and *SI Appendix*, Fig. S9 *D*–*F*. The times needed to reach 50% of the recorded maximum stomatal conductance reductions starting from 2 min after shifting to high VPD were calculated as half-response times.

### In-Gel Kinase Assays.

Transient Raf-M3Ks expression in mesophyll cell protoplasts and the following in-gel kinase assays were performed as described (54). In brief, 30 μg of mesophyll cell protoplasts isolated from 3- to 4-wk-old *Arabidopsis* leaves were transfected with 20 μg of pUC18 plasmids carrying *35S*:*Raf*-*M3K*-*GFP*:*nosT* using the polyethylene glycol-mediated method (107). After overnight incubation in incubation buffer (10 mM 2-[*N*-morpholino]ethanesulfonic acid [MES]-KOH, pH 6.0, 0.4 M mannitol, 20 mM KCl, and 1 mM CaCl_2_), protoplasts were harvested by centrifugation and lysed in sodium dodecyl sulfate–polyacrylamide gel electrophoresis sample buffer.

### Analyses of Guard-Cell Cytosolic Calcium Dynamics.

For analyses of cytosolic Ca^2+^ dynamics in guard cells, we used *A. thaliana* Col-0 plants expressing Yellow Cameleon YC3.60 under control of the *pGC1* promoter (58). Plants were grown on soil under long-day conditions (16/8 h at a photon fluence rate of 75 μmol·m^−2^·s^−1^ and a temperature of 20 °C). Small pieces of mature leaves of 4-wk-old plants were glued to the cover slide by using a medical adhesive (Hollister Inc.); upper cell layers were gently removed with a razor blade, thus isolating epidermal strips. The leaf epidermal strips were preincubated in a buffer solution (5 mM KCl, 10 mM MES, and 50 μM Ca^2+^, pH 6.15, adjusted with Tris base) for 2 to 3 h in white light (photon fluence rate of 125 μmol·m^−2^·s^−1^) to induce the stomatal opening to get turgid guard cells. The epidermal strips were then mounted on an open-top imaging chamber and perfused with the same solution (5 mM KCl, 10 mM MES, and 50 μM Ca^2+^, pH 6.15) as described (58, 108). To induce the hyperosmotic stress, after 5 min, the strips were perfused with the standard solution supplemented with 500 mM sorbitol. Epidermal strips were kept in this solution for 10 min, and then the hypertonic solution was replaced again with the standard buffer solution, without sorbitol, to induce the hypo-osmotic stress.

To perform Ca^2+^ imaging experiments, images of epidermises with stomatal guard cells were acquired with a Nikon TE300 inverted fluorescence microscope, with a FLUORESCENCE Module ILLUMINATOR (TE‐FM) epi‐fluorescence attachment (Nikon Inc.) using a Nikon 60× Plan Apo oil objective with a numerical aperture (n.a.) of 1.4. Excitation light was produced by a 75-W xenon fluorescent lamp (Osram) that was attenuated by 97% (3% light transmission) by using both 4× and 8× neutral density filters to reduce exposure of the fluorescent reporters and cells to epifluorescence excitation. Cameleon YC3.60 specific excitation and detection was achieved by using a yellow fluorescent protein/CFP filter set: 440/20 nm excitation, 485/40 nm emission for CFP, and 535/30 nm emission for cpVenus with a 455 nm dichroic long-pass filter (Chroma Technology). The filter wheel, shutter, and CoolSNAP charge-coupled device camera from Photometrics (Roper Scientific) were controlled by using Metafluor System software (MDS Inc.). Images were acquired every 3 s, on a time range of >30 min.

Fluorescence intensity was determined over regions of interest (ROIs) that corresponded to single guard cells. cpVenus and CFP emissions of the analyzed ROIs were used for the ratio (*R*) calculation (cpVenus/CFP). cpVenus/CFP ratios were normalized to the initial ratio (*R*_0_) and plotted vs. time (Δ*R*/*R*_0_). Background subtraction was performed independently for both channels before calculating the ratio. Data are representative of *n* = 6 experiments. *P* values were calculated with an unpaired Student's *t* test. Data are illustrated as scatter dot plots using GraphPad.

## Data Availability

All study data are included in the article and/or Dataset S1. Sequences of genes described in this article can be found in the *Arabidopsis* Genome Initiative database under the following accession numbers: *OST1*/*SnRK2.6* (AT4G33950), *SLAC1* (AT1G12480), *ALMT12*/*QUAC1* (AT4G17970), *OSCA1* (AT4G04340), *OSCA1.3* (AT1G11960), *OSCA1.7* (AT4G02900), *OSCA2.2* (AT1G10090), *OSCA2.3* (AT3G01100), *OSCA3.1* (AT1G30360), *GLR3.2* (AT4G35290), *GLR3.3* (AT1G42540), *CNGC5* (AT5G57940), *CNGC6* (AT2G23980), *CNGC19* (AT3G17690), *CNGC20* (AT3G17700), *CPK3* (AT4G23650), *CPK4* (AT4G09570), *CPK5* (AT4G35310), *CPK6* (AT2G17290), *CPK11* (AT1G35670), *CPK23* (AT4G04740), *CBL1* (AT4G17615), *CBL2* (AT5G55990), *CBL3* (AT4G26570), *CBL4* (AT5G24270), *CBL5* (AT4G01420), *CBL8* (AT1G64480), *CBL9* (AT5G47100), *MPK4* (AT4G01370), *MPK12* (AT2G46070), *M3Kδ1*/*RAF3* (AT5G11850), *M3Kδ3*/*EDR1*/*RAF2* (AT1G08720), *M3Kδ5*/*RAF6* (AT4G24480), *M3Kδ6*/*SIS8*/*RAF5* (AT1G73660), *M3Kδ7*/*RAF4* (AT1G18160), *CTR1*/*RAF1* (AT5G03730), *RAF16* (AT1G04700), *RAF40*/*HCR1* (AT3G24715), *RAF24* (AT2G35050), *RAF18* (AT1G16270), *RAF20* (AT1G79570), *RAF35* (AT5G57610), *RAF42* (AT3G46920), *RAF7* (AT3G06620), *RAF8* (AT3G06630), *RAF9* (AT3G06640), *RAF10* (AT5G49470), and *GHR1* (AT4G20940).
